# Saffron *(Crocus sativus)* petal as a new pharmacological target: a review

**DOI:** 10.22038/IJBMS.2018.31243.7529

**Published:** 2018-11

**Authors:** Azar Hosseini, Bibi Marjan Razavi, Hossein Hosseinzadeh

**Affiliations:** 1 Pharmacological Research Center of Medicinal Plants, Mashhad University of Medical Sciences, Mashhad Iran.; 2 Targeted Drug Delivery Research Center, Pharmaceutical Technology Institute, Mashhad University of Medical Sciences, Mashhad, Iran.; 3 Department of Pharmacodynamics and Toxicology, School of Pharmacy, Mashhad University of Medical Sciences, Mashhad, Iran.; 4 Pharmaceutical Research Center, Pharmaceutical Technology Institute, Mashhad University of Medical Sciences, Mashhad, Iran.

**Keywords:** Antidepressant, Crocus sativus, Hepatoprotective, Kaempferol, Metabolic syndrome, Saffron petal

## Abstract

Saffron petal is the main by-product of saffron processing which produced at high level but it is not applied and thrown out. Saffron petal is containing of several compounds such as mineral agents, anthocyanins, flavonoids, glycosides, alkaloids and kaempferol. As saffron petal is cheaper and produces in large amounts compared to saffron stigma, so, it can be considered as an appropriate source for different purposes. In this review different pharmacological properties of saffron petal such as anti-bacterial, anti-spasmodic, immunomodulatory, anti-tussive, anti-depressant, anti-nociceptive, hepato-protective, reno-protective, anti-hypertensive, anti-diabetic and anti-oxidant activity have been introduced. According to these properties, saffron petal can be used as an alternative or supplementary medicine in some diseases.

## Introduction

*Crocus sativus *(saffron) Linn. is belonging to Iridaceae family. It is used in foods as color and flavor agent and also used in cosmetic preparations (1). In comparison to other parts of plant, the stigma has more applications in food, cosmetic and treatment of diseases. Phytochemical studies have shown saffron stigma is containing crocetin, crocin, picrocrocin and safranal (2). The color of saffron is related to presence of crocin, while pharmacological properties is linked to crocetin (2). Also other components are found in saffron as flavonoids, anthocyanins, vitamins such as riboflavin and thiamine, proteins, starch, amino acids, mineral matter and gums (2). In traditional medicine, it is used as an aphrodisiac, antispasmodic, expectorant, stomachache, relieving tension, depression and insomnia. Also the powdered stigma of saffron was used in treatment of cataract. Other traditional applications are antibacterial, antiseptic and antifungal effects (3-5). In modern medicine other pharmacological properties of stigma including neuroprotective (6) antitussive, hypolipidemic (7, 8) anticonvulsant (9) antinociceptive (10) antidepressant (11) anxiolytic activity (12) cardiovascular protective (13) anticancer (14) and antioxidant (15, 16) have been reported. Saffron petal as a by-product is produced at high level but it is not used and thrown away after harvesting. However, it is worth to pay attention to the petal as it is cheaper than stigma. Based on evidences, most studies are about stigma of saffron and there is low information about saffron petal. In this review, we collected all studies about saffron petal properties and its pharmacological effects.

## Methods

This review was written according to finding data from scientific databases such as Scopus, Web of science, PubMed and local references which investigated different pharmacological properties of saffron petal. These data were collected through electronic databases from their inception to February 2018.

**Chemical compounds**

**Figure 1:**
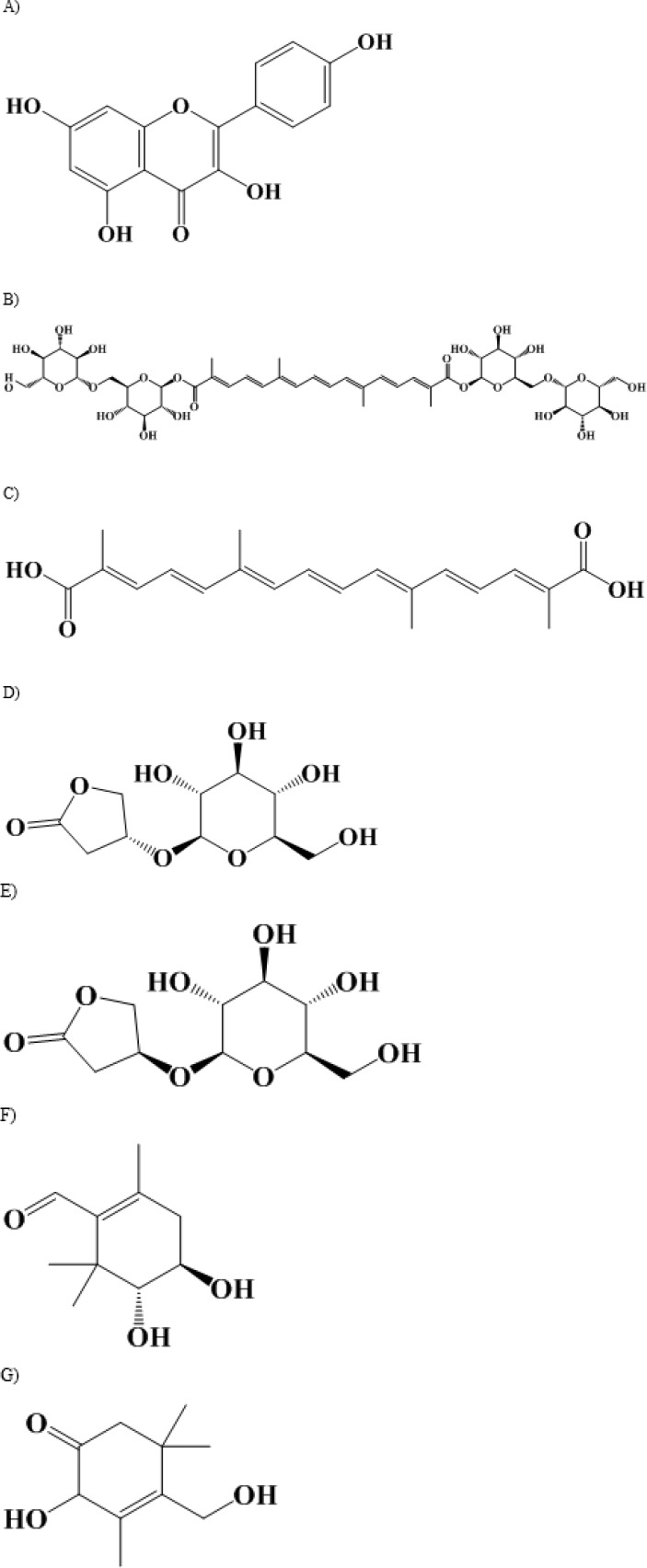
Chemical structures of some major bioactive constituents of saffron petal. A) kaempferol; B)crocin; C)crocetin; D) kinsenoside; E) goodyeroside A; F) crocusatin-K; G) crocusatin-L

Saffron petal is containing protein (10.20%), fat (5.3%), ash (7.00%), fiber (8.80%), sodium (25.75 mg/100 g), potassium (542.13 mg/100 g), calcium (486.25 mg/100 g), copper (0.87 mg/100 g), iron (17.99 mg/100 g), magnesium (2.93 mg/100 g), zinc (1.80 mg/100 g) and phosphorus (209.90 mg/100 g) (17). Also it is composed of flavonoles (kaempferol, 12.6%w/w) (18, 19) carotenoids (crocin, 0.6%w/w and crocetin) (19) anthocyanins (20) phenolic compounds (21) terpenoids and alkaloids (Table 1) (22) For first time, the HR-MAS NMR spectroscopy, showed the presence of kinsenoside, goodyeroside A and 3-hydroxy-- butyrolactone in intact saffron petal (23). The analysis of ethanolic extract of saffron petal by HR-NMR confirmed the presence of these compounds. In addition, the presence of kaempferol 3-*O-*3 sophoroside has been reported by “NMR-silent” in intact petals (23). The stigma and petal are containing terpenoids such as crocusatins with antityrosinase activity (24). Also, the petal includes anthocyanin which causes the purple color of saffron (24). Depending on pH, anthocyanins may be red, blue, or purple (25). The new monoterpenoids include crocusatin-J, 4-dihydroxybutyric acid were isolated from methanolic extract of saffron petal. Among the different isolated compounds, crocusatin-K, crocusatin-L, and 4-hydroxy-3,5,5-trimethylcyclohex- 2-enone show antityrosinase activity while protocatechuic acid, kaempferol, and kaempferol 7-*O*-*â*-D-glucopyranoside scavenge R,R-diphenyl-*â*-picrylhydrazyl (DPPH) radicals more than R-tocopherol (24) (Figure 1). 

**Saffron petal properties**

Nowadays, saffron petal is used as an organic agent in agriculture industries (26). The phytochemical studies have reported the presence of flavonoids and anthocyanins in saffron petal which showed beneficial effects as supplementary compounds (19). In traditional medicine, saffron petal is consumed as antispasmodic, stomachic, curative of anxiety, antitumor and antidepressant. According to economical properties, phytochemical compounds and traditional usage, it can be used in different medicinal fields (27). 

**Pharmacological Properties**

**Antibacterial**

**Figure 2:**
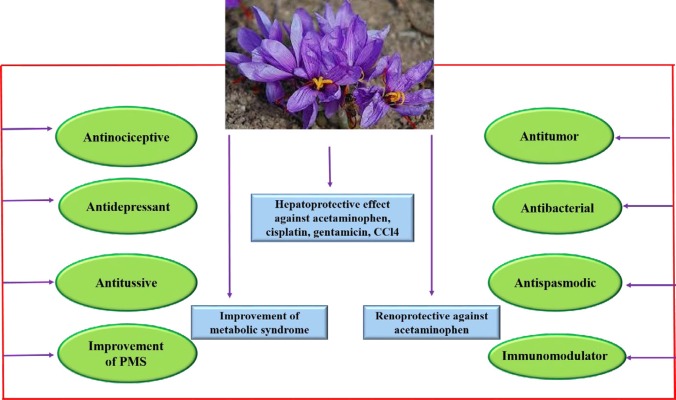
The pharmacological effects of saffron petal

Abbreviation: Tetrachloride carbon (CCl4), premenstrual syndrome (PMS)

Food poisoning is caused via eating contaminated foods, toxic plants, fungi or animal materials via entering of bacteria to body. However, using of anti-microbial agents or preservatives can be effective in prevention of bacterial growth (28). The studies have shown some of natural products such as essential oils, herbs and spices have anti-microbial or anti-fungal properties, therefore can be used as an anti-microbial agents (29, 30). The methanolic extract of saffron petal showed anti-bacterial activity against* Staphylococcus* *aureus, Bacillus cereus, Salmonella typhi, Escherichia coli *and *Shingella dysenteriae* at concentration of 1000 mg/ml, with inhibition zone diameters ranging from 13 to 22 mm. The ethyl acetate extract prevented the growth of *B. cereus *with inhibition zone diameter of 15 mm. The water and chloroform extracts had lesser activity against mentioned strains. Based on this study, methanolic, ethyl acetate and aqueous extracts exhibited anti-microbial activity against *S. dysenteriae* (Table 2, Figure 2) (31).

**Antispasmodic effects**

The rat isolated vas deferens and guinea pig isolated ileum were used to investigate effect of saffron petal on tonicity of smooth muscle. The petal extracts reduced electrical field stimulation (EFS)-induced contraction in rat isolated vas deferens. In rat isolated vas deferens, petal extracts reduced responses to epinephrine. However, Fatehi et al., showed that petal extract antagonized the adrenergic receptors of rat isolated vas deferens. Also, EFS induced contraction in guinea-pig isolated ileum through muscarinic receptors. The petal extract decreased EFS-induced contraction via inhibition of muscarinic receptors (Table 2, Figure 2) (32).

**Immune System**

The most of herbal medicines have immunomodulatory effects and alter immune function. The role of herbal medicines in modulation of cytokine secretion, histamine release, immunoglobulin secretion, cellular co-receptor expression, lymphocyte activation and phagocytosis has been reported in different studies (33-35)). In a study conducted on rats received saffron petal extract at doses of 0, 75, 150, 225, and 450 mg/kg for 14 days, no difference between treated groups with control in hematological parameters such as red blood cells, hemoglobin, hematocrit, and platelet has been observed. Saffron petal extract increased IgG at dose of 75 mg/kg in comparison with other groups. No damage was shown in spleen according to the results of pathology. This study showed that saffron petal had immune-stimulatory effect at dose of 75mg/kg (Table 2, Figure 2) (36).

**Premenstrual syndrome (PMS)**

In a double blind clinical study, the women (20-45years) who experienced PMS symptoms for at least 6 months, received saffron petal twice a day (15 mg/kg at morning and 15 mg/kg at evening). The control group received placebo capsule twice a day. The protocol was done for two menstrual cycles (cycles 3 and 4). The results showed saffron petal improved PMS in comparison with control group (Table 2, Figure 2) (37).

**Antitussive activity**

The antitussive activity of *C.* *sativus* (stigma and petal) and it active ingredients (safranal and crocin) was investigated in guinea pigs using nebolized solution of citric acid 20%. The agents were injected intraperitoneally. The ethanolic extract of *C. sativus *at doses of 100‐800 mg/kg and safranal at doses of 0.25‐0.75 ml/kg decreased the number of coughs significantly. The ethanolic (200, 400, 800mg/kg) and aqueous (80, 160, 320mg/kg) extracts of petal and crocin (50, 200, 600 mg/kg) did not improve cough (Table 2, Figure 2) (7).

**Antidepressant effects**

Depression is a psychological disorder which influences thought, behavior and mood. The depressed persons miss their hope and energy for doing of activities. The uncontrolled of depression may lead to suicide. However, the treatment of depression is important. There are different ways for the treatment but using of anti-depressant drugs are the most common. These drugs influence the level of neurotransmitters in brain. Whereas, these drugs have positive effects but can cause side effects in long term. Nowadays, the studies have reported that herbal medicine can be effective in mood disorders (38). Different studies have reported saffron plays an important role in modulation of mood (39). A double-blind randomized, placebo-controlled trial showed consumption of saffron stigma (15mg/Bid for 8 weeks) reduced the symptoms of postpartum depression in breast feeding mothers (40). Also, antidepressant activity of aqueous and ethanolic extracts of saffron stigma, safranal and crocin were evaluated in mice by force swimming test. The immobility time reduced by saffron stigma (0.0.8g/kg), safranal (0.15-0.5ml/kg) and crocin (50-600mg/kg). Safranal and both extracts of stigma increased swimming time (11). In another study, male Wistar rats received crocin at doses of 12.5, 25 and 50 mg/kg for 21 days. The immobility time in force swimming test reduced by crocin. Also crocin at doses of 25 and 50 mg/kg, increased the expression of brain-derived neurotrophic factor (BDNF) and cAMP response element binding protein (CREB) in hippocampus. Crocin at all doses increased the level of VGF (41). However, anti-depressant activity of crocin may be related to increase of CREB, VGF and BDNF (41). Also, in other study, rats received aqueous extract of saffron at doses of (40, 80 and 160mg/kg/day) for 21 days. Results showed, saffron reduced immobility time in forced swimming test. Also, saffron increased BDNF and CREB in hippocampus (42). A double-blind, randomized and placebo-controlled trial showed the efficacy of *C. sativus* petal in the treatment of mild‐to‐moderate depression for 6‐weeks. In this study, the patients received *C. sativus* petal as capsule at dose of 30 mg/day (BID) for 6‐weeks. While control group received placebo capsule for the mentioned time. After 6 weeks, according to the Hamilton Depression Rating Scale, *C. sativus *petal showed better antidepressant effect than placebo (43). In another clinical study the antidepressant effect of saffron petal was similar to fluoxetine (44). The adverse effects were not observed in both groups. However, the petal extract plays an effective role in treatment of mild to moderate depression (43). In comparison with saffron stigma, the saffron petal is cheaper, however using of petal as an anti-depressant agent, can be appropriate economically. Moreover, the anti-depressant activity of kaempferol, an active compound of saffron petal, was investigated in mice and rats by using forced swimming test. Kaempferol was injected intraperitoneally in mice (100 and 200 mg/kg) and rat (500 mg/kg) and compared with fluoxetine as a positive control (20 mg/kg). Kaempferol reduced immobility time in mice similar to fluoxetine (45). (Table 2, Figure 2).

**Antinociceptive and anti-inflammatory effects**

C. sativus and its constituent safranal have shown preventive effects on serum inflammatory markers in sensitized guinea pigs (46-48).The anti-nociceptive and anti-inflammatory effects of ethanolic and aqueous extracts of saffron petal were investigated in mice. The results showed both of extracts had anti-nociceptive effects against chemical-induced pain. Also, the ethanolic extract reduced chronic inflammation and did not affect on acute inflammation. The observed effects may be related to presence of compounds such as flavonoids, tannins, anthocyanins, alkaloids, and saponins (Table 2, Figure 2) (10).

**Hepatoprotective**

**Carbon tetrachloride (CCl4)**

Carbon tetrachloride is a toxic agent for liver and leads to injuries via fatty degeneration, cellular necrosis, fibrosis and cirrhosis (49). Anti-oxidants and free radical scavengers can protect liver cells against chemical-induced hepatotoxicity (50). The aqueous extract of petal was administrated at dose of 1 g/kg after 1 and 6h of CCl4 injection. The levels of alanine aminotransferease (ALT) and aspartate aminotransaminase (AST) decreased following treatment by ethanolic and aqueous extracts of petal. Also, the histopathological studies showed the petal extracts reduced liver lesions induced by CCl4. Antioxidant properties of petal reduced the function of cytochrome P450 for generation of CCl4 metabolites as free radicals (Table 2, Figure 2) (51).

**Acetaminophen**

Acetaminophen is used commonly as an analgesic and antipyretic drug. Acetaminophen is metabolized and converted to N-acetyl-p-benzoquinone imine (NAPQI) by cytochrome P450 enzymes (52). At therapeutic doses of acetaminophen, the level of produced metabolite is small but at overdose, liver generates high level of NAPQI (53). The high production of NAPQI causes the depletion of glutathione and liver injuries. Oxidative stress happens in hepatic cells following reduction of glutathione (54). The rats were pretreated with *C. sativus *petal at doses of 10 and 20 mg/kg for 6 days, then acetaminophen was administrated orally at dose of 600 ma/kg. Following acetaminophen injection, the amount of AST, ALT and bilirubin increased, while total protein and albumin reduced. *C. sativus* petal at dose of 20 mg/kg restored the acetaminophen toxicity by reduction of AST, ALT and bilirubin levels and improved serum albumin values. The pathological injuries observed in acetaminophen group were cell swelling, severe inflammation and necrosis, while, *C. sativus* petal led to mild injury at high dose (Table 2, Figure 2) (55).

**Gentamicin**

One type of liver injury is identified by blood-filled cavities. Some of diseases including AIDS, tuberculosis, cancer and consumption of drugs such as anabolic steroids and azathioprine cause the above problem (56). A study investigated the protective effect of saffron petal against gentamicin-induced peliosis hepatis in rats. The rats received gentamicin at dose of 80mg/kg for 7 days. Saffron petal was administered at doses of 40 and 80 mg/kg for 7 days. Neither doses of saffron could reduce peliosis hepatic and telangiectasis induced by gentamicin (Table 2, Figure 2) (57).

**Cisplatin**

Cisplatin as a chemotherapeutic drug induces hepatotoxicity. The side effect of cisplatin is related to oxidative stress and production of ROS, which damages cell membrane. Also, anti-oxidant enzymes reduced cisplatin injury (58) (59). In a study, the rats were received cisplatin (0.4 mg/kg) for 8 weeks and silymarin as well as hydro-alcoholic saffron petal (40 and 80 mg/kg) were gavaged for 8 weeks. Cisplatin reduced anti-oxidant enzymes, increased malondialdehyde (MDA) and led to liver injury. The extract and silymarin decreased AST, ALT, MDA and bilirubin levels while increased total protein and albumin levels in serum. Silymarin and hydro-alcoholic saffron petal reduced the toxicity of cisplatin via anti-oxidant properties (Table 2, Figure 2) (60).

**Renoprotective**

**Acetaminophen**

In a study, acetaminophen was injected to rats at dose of 600 mg/kg. Also, saffron petal extract was administered at doses of 10 and 20 mg/kg for 8 days. Acetaminophen increased creatinine and uric acid levels as well as pathological changes in renal. While the extract at high dose reduced renal toxicity via reduction of uric acid and creatinine levels (Table 2, Figure 2) (61).

**Table 1:** The amounts of active ingredients in saffron petal

Compound	Amount
Protein (17)	10.20%
Fat (17)	5.3%
Ash (17)	7%
Fiber (17)	8.80%
Sodium (17)	25.75 mg/100 g
Potassium (17)	542.13 mg/100g
Calcium (17)	486.25 mg/100 g542.13 mg/100 g
Copper (17)	0.87 mg/100g
Iron (17)	17.99 mg/100g
Magnesium (17)	2.93 mg/100g
Zinc (17)	1.80 mg/100g
Phosphorus zinc (17)	209.90 mg/100g
Kaempferol (18)	12.6%w/w
Crocin (19)	0.6%w/w
Anthocyanins (20)	1712 mg/L extract
Phenolic compounds (21)	3.42mg
Terpenoids (21)	-
Alkaloids (17)	-

**Table 2:** The pharmacological effects of *C. sativus* petal in *in vitro*, *in vivo* and clinical studies

Effect	Study design	Dose/duration of study	Results	Ref.
Antibacterial	*In vitro*	Chloroform, ethyl acetate, methanolic and water extracts were used against different strains at doses of 31.2, 62.5, 125, 250, 500 and 1000 mg/ml	Methanol, ethyl acetate and aqueous extracts inhibited the growth of bacteria with different potencies. The effect is related to presence of phenolic compounds and anti-oxidant activity	(31)
Muscle relaxant	*In vitro*	EFS-induced contraction in isolated vas deferens and guinea-pig ileum at dose of 560 mg/ml	↓contractility in both of preparations via blocking of postsynaptic receptors. It can be related to antagonistic effect of the aqueous extract on adrenergic receptors of rat isolated vas deferens	(32)
Immuno stimulator	*In vivo (rat)*	0, 75, 150, 225, and 450 mg/kg for 14 days.	No change on RBC, Hb, Hct, and platelet,↑ IgG at dose of 75 mg/kg	(36)
Premenstrual syndrome (PMS)	human	15 mg/kg at morning and 15 mg/kg at evening	Improvement of PMS, may be due to effect on serotonergic system	(37)
Antihypertensive	*In vivo(rat)*	50mg/100g	↓ MABP	(32)
Antitussive	*In vivo*	The ethanolic extract of *C. sativus *( 100‐800 mg/kg), safranal ( 0.25‐0.75 ml/kg), ethanolic (200, 400, 800mg/kg) and aqueous (80, 160, 320mg/kg) extracts of petal and crocin (50, 200, 600 mg/kg) were injected intraperitoneally with nebulized solution of citric acid 20% in guinea pigs	↓Number of coughs by ethanolic extract and safranal. The ethanolic and aqueous extracts of petal and crocin did not improve cough	(7)
Antidepressant	Human	at dose of 30 mg/day (b.d.) for 6‐weeks	↓signs of depression maybe due to increase the level of serotonin	(43, 44)
	human	saffron stigma (15mg/Bid for 8 weeks) on postpartum depression in breast feeding mothers	↓symptoms	(40)
	*In vivo*(mice and rat)	Kaempferol	↓Immobility time in mice and rat	(45)
	*In vivo*(mice)	saffron stigma (0.0.8g/kg), safranal (0.15-0.5ml/kg) and crocin (50-600mg/kg)	↓Immobility time by saffron stigma, safranal and crocin. ↑swimming time by safranal and extracts of stigma may be due to uptake inhibition of dopamine, orepinephrine and serotonin	(11)
	*In vivo*	crocin (12.5, 25 and 50 mg/kg) for 21 days in rats.	↓ immobility time ↑expression of BDNF and CREB and VGF in hippocampus	(41)
Antinociceptive and antiinflammatory	*In vivo*	Saffron petal	↓pain induced by chemical compounds and chronic inflammation.	(10)
Antidyslipidemia	*In vivo*	stigma (40mg/kg), petal (80 mg/kg) and combination (80 mg/kg) for 3 weeks	↓TG, LDL, leptin, insulin resistant, AST, ALT and ALP. LDL/HDL and TC/HDL	(74, 76)
Antidiabetic	*In vivo*	100 or 200 mg/kg, orally, in STZ-diabetic rats for 28 days	↓FBS and BUN without changing Cr. through reducing extracellular matrix accumulation and its antioxidant properties.	(76)
		**Hepatoprotective **		
Carbon tetrachloride	*In vivo*	1 g/kg after 1 and 6h after CCl4 injection	↓AST and ALT improve anti-oxidant enzymes ↓ liver lesion. may be due to antioxidant activity and radical scavenging, reduction of CCl4 metabolic activation by cytochrome P450 inhibition and fixation of hepatic cell membrane.	(51)
Acetaminophen	*In vivo*	10 and 20 mg/kg for 6 days	↓AST, ALT, bilirubin and improvement of albumin due to anti-oxidant activity	(55)
Gentamicin	*In vivo*	40 and 80 mg/kg for 7 days.	could not reduce peliosis hepatic and telangiectasis induced by gentamicin	(57)
Cisplatin	*In vivo*	hydroalcoholic saffron petal (40 and 80 mg/kg) were gavaged for 8 weeks.	↑total protein and albumin, ↓AST, ALT, bilirubin and MDA levels related to anti-oxidant properties	(60)
		**Renal protective**		
Acetaminophen	*In vivo*	10 and 20 mg/kg for 8 days	↓uric acid, cr and renal injury at high dose	(61)

Red blood cell (RBC), Hemoglobin (Hb), Hematocrit (Hct), Alanine Aminotransferease (ALT), Aspartate transaminase (AST), Alkaline phosphatase (ALP), Fast blood sugar (FBS), Blood urea nitrogen (BUN), Total cholesterol (TC), Triglyceride (TG), Molondialdehyde (MDA), Low density lipoprotein (LDL), High density lipoprotein (HDL) and Mean arterial blood pressure (MABP), brain-derived neurotrophic factor (BDNF), cAMP response element binding protein (CREB) and creatinine (Cr).

**Metabolic Syndrome**

The metabolic syndrome is complex of problems including diabetes, cardiovascular problems, obesity, nonalcoholic fatty liver disease and kidney dysfunction (62). Different studies have shown herbal medicine play role in reduction of metabolic syndrome symptoms. For example natural products such as *Garcinia mangostana *(63), *Berberis vulgaris *(64), *Camellia sinensis* (green tea) (65) *Persea americana* (Avocado) (66), *Cinnamomum verum* (Cinnamon) (67), *Rosmarinus officinalis ***(**Rosemary) (68), *Vitis vinifera *(Grape) (69), *Allium sativum* (70) and *Nigella sativa* L. (71). Recent studies have shown, saffron and its constituents have protective effects on metabolic syndrome (72). Different studies have reported therapeutic effects of saffron petal on risk factors of metabolic syndrome such as obesity, hypertension and diabetes. 

**Antihypertensive activity**

The effect of *C. sativus *petal extract was investigated on blood pressure in anesthetized rats. The aqueous and ethanolic extracts of *C. sativus *petal extract reduced blood pressure at dose of 50 mg/100g. The reduction of blood pressure could be related to effect of *C. sativus* on heart or peripheral resistance. In this study administration of extract did not change hear rate. However, results showed the effect of extract on peripheral resistance is important factor in decrease of blood pressure (Table 2, Figure 2) (32). 

**Antiobesity and Antidyslipidemia**

Obesity is an epidemic disease in worldwide. The chronic obesity is an important factor for metabolic syndrome. The obesity is accompanied with hypertension, insulin resistance, and hyperlipidemia (73). In a study the effect of saffron petal and stigma were investigated in overweight rats. The rats were received high-fat diet for 10 weeks. Then saffron stigma (40 mg/kg), petal (80mg/kg) and combination of them (80mg/kg) were gavaged to rats for 3 weeks. The results showed the extracts decreased total cholesterol, triglyceride and LDL, while, increased HDL levels. Also the extracts reduced atherosclerosis-index (LDL/HDL), atherogenic index (TC/HDL), and the liver enzymes including ALT, AST and alkaline phosphatase (ALP). The levels of leptin and insulin were reduced by saffron extracts. Results indicated that saffron extracts enhanced anti-oxidant level, while, reduced lipid peroxidation. However, the extracts ameliorated dyslipidemia in obese rats via reduction of atherosclerosis and insulin resistant (Table 2, Figure 2) (74).

**Antidiabetic**

Streptozotocin (STZ) is a toxic compound for pancreatic β cells. This compound damages pancreatic β cells and leads to lower insulin level and elevates blood glucose (75). In STZ-diabetic rats, saffron petal extract was given orally at doses of 100 or 200 mg/kg for 28 days. STZ increased fasting blood sugar (FBS), urine volume, blood urea nitrogen (BUN), and creatinine (Cr) levels. The extract at dose of 200 mg/kg reduced FBS, while, urine volume and BUN level decreased by both of doses. The level of Cr was not changed by saffron petal. Also the extract improved the histological damages induced by STZ. According to this study, extract protected against STZ-induced nephropathy (Table 2, Figure 2) (76).

**Antioxidant activity**

Reactive oxygen species lead to different diseases via formation of superoxide anion radical, hydroxyl radical, and hydrogen peroxide, which damage cell membrane and attack molecules such as DNA, protein, lipids and small cellular molecules (77). Most of herbal medicines are containing anti-oxidant compounds which scavenge free radicals and reduce cellular damage. A study showed antioxidant activity of saffron petal in lambs using the 2, 2-diphenyl-1-picrylhydrazyl (DPPH) free-radical method. The extracts of saffron petal were gavaged at doses of 500, 1000 and 1500 mg/kg for 15 days. At last day of trial, results showed that saffron petal increased anti-oxidant content at all doses. The extract did not change the levels of glucose, uric acid, creatinine, AST, ALT, ALP, MDA, total thiol, BUN and other indexes (Table 2) (78).

**Antitumor activity**

Anti-tumor activity of saffron stigma and petal was evaluated using brine shrimp and potato disk. Results showed that the IC50 values of saffron extracts were 5.3mg/ml and 10.8 mg/ml for petal and stigma extracts against tumor, respectively (Table 2, Figure 2) (79).

**Toxicity of saffron petal**

According to toxicological studies, toxicity of stigma is more than petal. Study reported that consumption of 1.2 g saffron led to diarrhea, bleeding, nausea and vomiting (3). For determination of LD_50_, different doses of saffron stigma and petal were injected to rats intraperitoneally (i.p.) and the mortality was evaluated after 24h. The LD50 values of saffron stigma and petal in mice were 1.6 and 6 g/kg, respectively (80). In a sub-acute toxicity study, saffron stigma was injected i.p. at doses of 0.16, 0.32 and 0.48 g/kg, while, petal was administrated at doses of 1.2, 2.4 and 3.6 g/kg for two weeks. This study reported that saffron petal and stigma extracts reduced body weight, hematocrit, hemoglobin and erythrocytes. Pathological examination showed stigma did not cause damage in different organs significantly, while, liver and lung injuries were observed in animals received saffron petal (80).

## Conclusion

This review showed that saffron petal is composed of different active ingredients such as anthocyanins, flavonoles (kaempferol), new monoterpenoids include crocusatin-J and 4-dihydroxybutyric acid. Among the different isolated compounds, crocusatin-K, crocusatin-L, and 4-hydroxy-3,5,5-trimethylcyclohex- 2-enone show antityrosinase activity while protocatechuic acid, kaempferol, and kaempferol 7-*O*-*â*-D-glucopyranoside scavenge R,R-diphenyl-*â*-picrylhydrazyl (DPPH) radicals more than R-tocopherol. Saffron petal is cheaper and produces in large amounts compared to saffron stigma, so, it can be considered as an appropriate source for different purposes. It has different pharmacological effects such as anti-bacterial, hepatoprotective, renoprotective, antidiabetic, antihypertensive, antidyslipidemia, antidepressant, antioxidant and antitumor properties. The most of pharmacological effects is related to the presence of active components in saffron petal which most of them exhibit anti-oxidant activities. According to this review, saffron petal can be used as an alternative or supplementary drug in medicine. Of course, it should be noted that the most of studies which mentioned in this review are animal and clinical studies are low. However it is necessary to do more human studies. 

## Declaration of Conflicting Interests

The authors declare not to have any conflicts of interest.
